# Ordered and ultrathin reduced graphene oxide LB films as hole injection layers for organic light-emitting diode

**DOI:** 10.1186/1556-276X-9-537

**Published:** 2014-10-01

**Authors:** Yajie Yang, Xiaojie Yang, Wenyao Yang, Shibin Li, Jianhua Xu, Yadong Jiang

**Affiliations:** 1 State Key Laboratory of Electronic Thin Films and Integrated Devices, School of Optoelectronic Information, University of Electronic Science and Technology of China (UESTC), Chengdu 610054, People's Republic of China

**Keywords:** Reduced graphene oxide, Conducting polymer, LB films, OLED, Hole injection layer

## Abstract

In this paper, we demonstrated the utilization of reduced graphene oxide (RGO) Langmuir-Blodgett (LB) films as high performance hole injection layer in organic light-emitting diode (OLED). By using LB technique, the well-ordered and thickness-controlled RGO sheets are incorporated between the organic active layer and the transparent conducting indium tin oxide (ITO), leading to an increase of recombination between electrons and holes. Due to the dramatic increase of hole carrier injection efficiency in RGO LB layer, the device luminance performance is greatly enhanced comparable to devices fabricated with spin-coating RGO and a commercial conducting polymer PEDOT:PSS as the hole transport layer. Furthermore, our results indicate that RGO LB films could be an excellent alternative to commercial PEDOT:PSS as the effective hole transport and electron blocking layer in light-emitting diode devices.

## Background

The two-dimensional (2D) single-layer carbon material, graphene, has emerged as a rising star in the field of materials [1]. Owing to its unique electrical, chemical, and mechanical properties [2], graphene has been developed for various applications in optoelectronics [3], sensors [4,5], and electrochemistry [6,7]. Meanwhile, many studies on graphene-based photovoltaic applications have been carried out, in which graphene was used as active layer materials, electron transport bridges, and transparent electrodes [8-10]. However, the general approaches used to prepare graphene, for instance CVD thermal evaporation, result in high cost fabrication process [11,12].

As the surging interest in graphene-based materials, graphene oxide (GO) has regained significant attention as a solution-processable precursor for bulk production of graphene used on transparent conductors and supercapacitors [13]. The reduced graphene oxide (RGO) can be obtained by reducing GO through chemical and thermal treatment [14]. It has been also demonstrated that RGO exhibits high mechanical strength, as well as combined with interesting physical properties, including high performance of electrical, thermal conductivity, and electrochemical activity [15-17].

Due to the high transparent and electrical performance, RGO has been utilized as an electrode layer for optoelectronic devices such as organic light-emitting diode (OLED) and organic photovoltaic devices [18,19]. RGO film deposition methods, such as spin-coating, spray coating etc., have been demonstrated as effective methods to deposit RGO on indium tin oxide (ITO) electrode as a hole injection layer [20,21]. However, it is still a challenge to address ordered and thickness-controlled arrangement of RGO on ITO as high performance hole injection layers. The arrangement feature of carbon sheets results in the remarkable change of electrical ability that influences the performance of OLED devices dramatically. So, it is worthwhile to obtain well-arranged RGO sheets on ITO as hole injection layer, which provide a superior hole injection performance to prepare OLED devices with high luminescent efficiency.

It is well known that GO can float on a water surface without the need for surfactants or stabilizing agents and some reports about the Langmuir-Blodgett (LB) deposition of GO films have been reported [22,23], but few works focused on the optoelectronic electrode applications of these GO-based LB films. In our previous work, a RGO/conducting polymer composite was prepared as high performance electrochemical capacitor electrode [24]. In this paper, we demonstrate the preparation of a well-ordered and thickness-controlled RGO layer on ITO surface as hole injection layer by using the LB method. This RGO hole injection layer offers tunable arrangement and loading of RGO on a substrate. Limited work is focused on fabricating RGO LB layers as hole injection layer and the performance of related device. Owing to the flexible nature of RGO, the LB deposition technique can substantially suppress the folding and wrinkling of graphene oxide sheets, and the sheets are able to be transferred onto a substrate with defined structure, which could provide preferred film structure for effective hole carrier injection.

## Methods

### Materials

Graphite flakes used for GO preparation were purchased from Sigma-Aldrich (St. Louis, MO, USA). GO was synthesized from natural graphite flakes through Hummer's method [25]. The size of graphite flakes was 380 μm (grade 3061). In order to obtain stable GO dispersion for LB deposition, 20 mg GO was dissolved in 80 ml methanol/deionized (DI) water (volume ratio 4:1) mixture solution, and the solution was subjected to ultrasonication for 30 min followed by centrifugation at 2,500 rpm. N,N′-Bis(3-methylphenyl)-N,N′-diphenylbenzidine (TPD) and Tris(8-hydroxyquinolinato)aluminium Alq3 were also purchased from Sigma-Aldrich. A commercial conducting polymer PEDOT:PSS (product code as Clevios™ P Jet) was purchased from Bayer company (Leverkusen, Germany). Aluminum as a cathode was purchased from Dongyang Inc. (Shenzhen, China). All solvents used in experiment are high purity level.

### Film and device fabrication

The preparation of different-layer GO sheets was carried out in a KSV-5000 LB system (KSV Instruments Ltd., Helsinki, Finland). The self-assembly performance of GO at air-water interface was characterized by a BAM-300 Brewster angle microscopy (KSV Instruments Ltd., Helsinki, Finland). Before the film preparation, the trough was carefully cleaned with chloroform and then filled with DI water. GO solution was dropwise spread onto the water surface by using a glass syringe. Surface pressure was monitored through a tensiometer attached to a Wilhelmy plate. The film was compressed by the barriers at a speed of 1 mm/min. The GO monolayers were transferred to substrates at various points during the compression by vertically dipping the substrate into the trough and slowly pulling it up (1 mm/min). The substrate was first processed with a hydrophilic treatment in order to deposit uniform GO LB layers. By repeating this vertical dipping process, different-layer GO sheets were deposited on substrate uniformly with a layer-by-layer structure. After the deposition of GO LB films, the substrate was treated in a water vapor oven at 200 C^o^ for 6-h GO reduction. Surface morphology of GO and RGO films were investigated by SP 3800 atomic force microscopy (AFM; Seiko Instrument Industry Corporation, Tokyo, Japan) with a tapping mode. The morphological properties of GO and RGO were also investigated with Hitachi S-2400 scanning electron microscopy (SEM; Hitachi, Ltd., Chiyoda, Tokyo, Japan). UV-vis spectrum of the film was recorded on a UV 1700 spectrometer (Shimadzu Corporation, Nakagyo-ku, Kyoto, Japan).

After the preparation of RGO on substrate, the following active layers for OLED were prepared through spin-coating method. The TPD and Alq3 solutions were spin-coated on RGO-covered substrate at 2,000 rpm. The devices used a 50-nm TPD as the hole transport layer and 50-nm Alq3 as the electron transport layer. After the deposition of active layer, an Al cathode electrode was deposited onto the active layer by thermal evaporation in vacuum with a thickness of 60 nm. The OLED with a structure of ITO/RGO LB films/TPD/Alq3/Al-electrode was fabricated, and the device performance was measured at room temperature. For electrical performance testing of GO and RGO films, A Si/SiO_2_ substrate with prepatterned electrodes was used for current-voltage (I-V) measurement. The interdigitated electrodes had a 15 and 30 μm channel length. The I-V curves were characterized by a Keithley 4200 semiconducting testing system (Cleveland, OH, USA).

## Results and discussion

**Figure 1:**
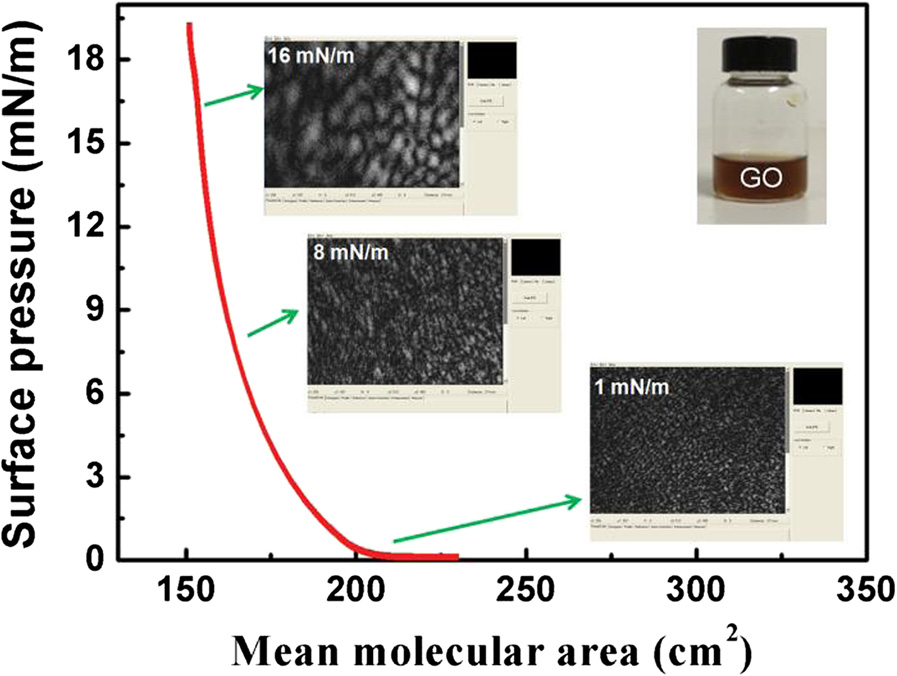
**Surface pressure-mean molecular area (π-A) isotherm curves of GO at air-water interface.** The inset images show the BAM image of GO evolution at different surface pressures.

**Figure 2:**
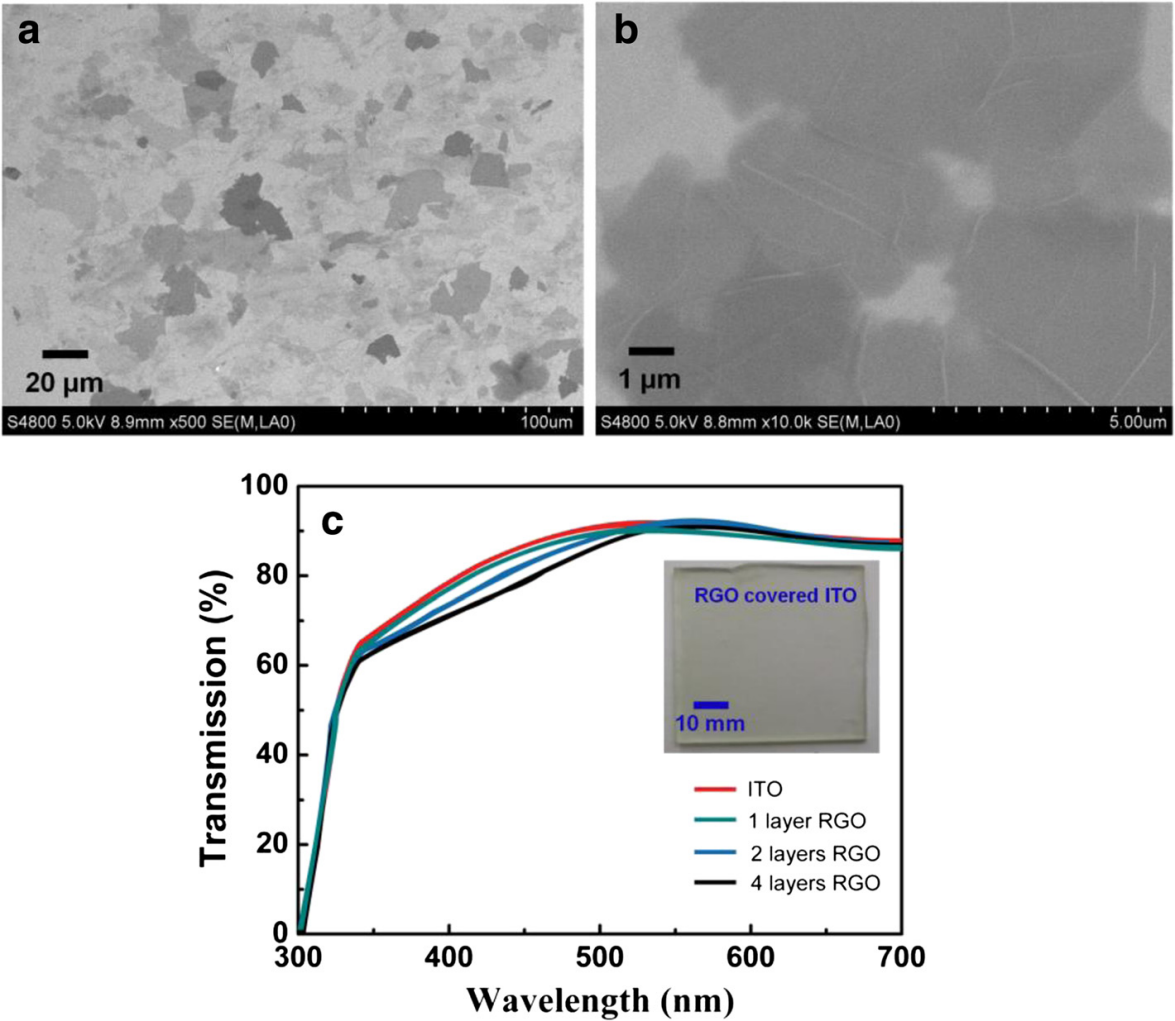
**SEM images of GO LB films and transmission performance of RGO LB film. (a-b)** SEM images of GO LB films with different magnifications after thermal reduction and **(c)** the transmission performance of RGO LB film-covered ITO.

GO can float on a water surface without surfactants or stabilizing due to the geometric similarity of GO with air-water interface, which makes it ideal for accommodating the flat sheets. It can be seen from Figure 1 that GO sheets arrange compactly at the air-water interface with continuous compression. This compact arrangement of GO sheets can be constructed on different substrates through vertical or horizontal deposition. By controlling the surface pressure, the uniform and compact arrangement of graphene oxide sheets can be obtained. Moreover, large area LB deposition can also be realized on GO sheets with tunable arrangement on different substrates.Due to the low conductivity, pure GO is not suitable as a carrier transportation layer and a further-reduced treatment on GO leads to formation of RGO with better conductive performance. Figure 2 shows SEM images of two-layer GO sheets deposited on Si substrate after the thermal treatment. The results reveal that the thermal treatment does not lead to distinct morphology change, and the RGO sheets obtained from GO LB films keep the ordered and compact formation. An investigation of surface roughness characterized by AFM indicates that LB deposition of RGO on ITO gives rise to the surface roughness decrease of ITO. The average thicknesses of the 1-, 2-, and 4-layer RGO LB films were estimated to be 1.2, 2.1, and 3.5 nm, respectively, corresponding to roughness (rms) of 0.50, 0.88, and 1.76 nm. These roughness values are lower than the bare glass/ITO substrate, indicating that the deposition of RGO layers serves to planarize the anode surface. The uniform RGO LB layers display excellent covering performance as the hole injection layer between ITO and active layer. Figure 2c shows the transmission performance of ITO covered by different-layer RGO films. Although the transmittance decreases slightly with thickness, the optical transmission spectra reveal that the GO thin films do not significantly change the transparency of the ITO electrode.

**Figure 3:**
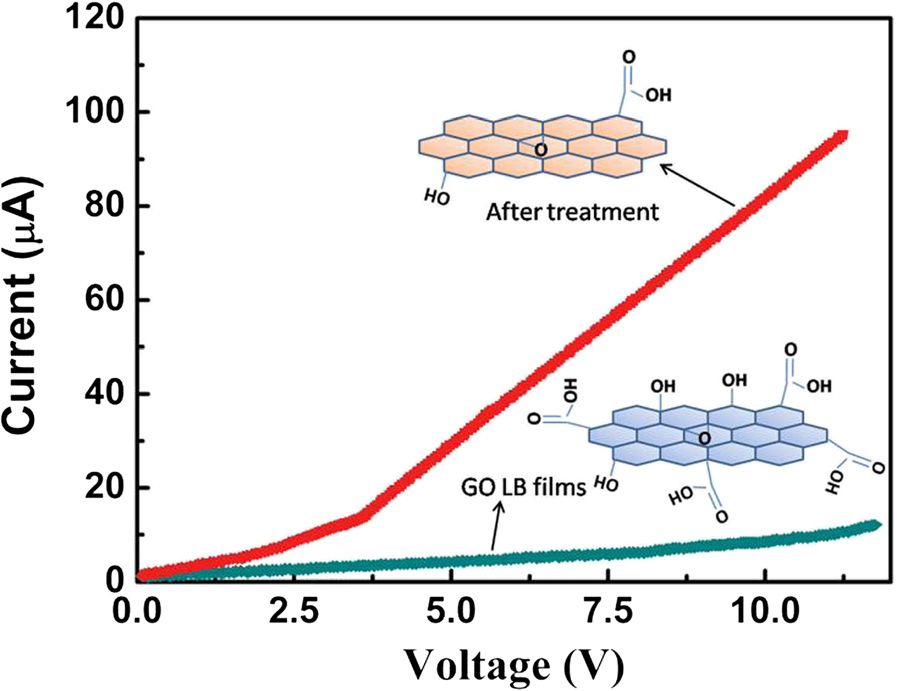
Current versus voltage (I-V) performance of GO and RGO.

**Figure 4:**
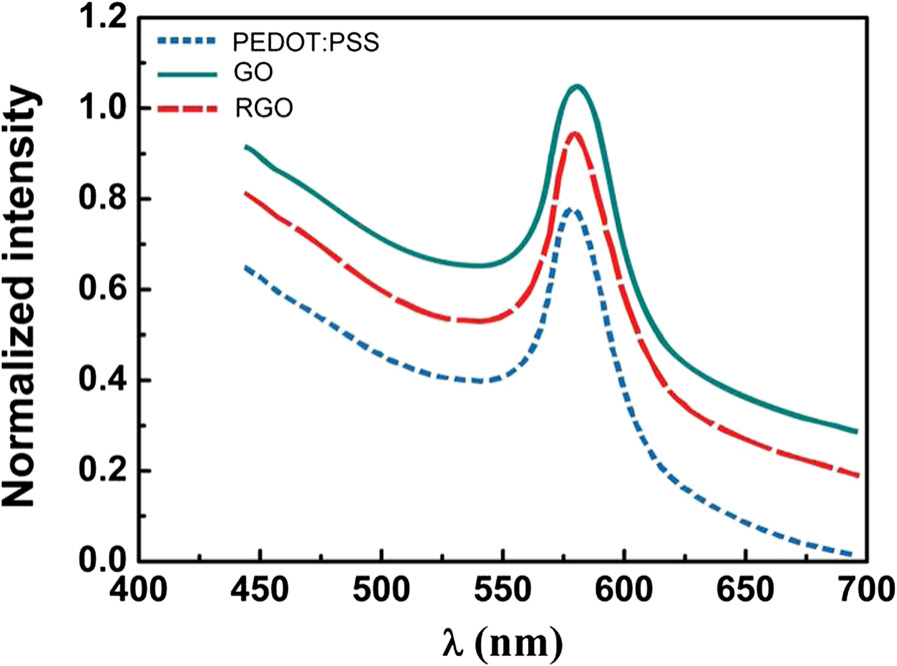
EL spectra of devices with different films as hole injecting layer.

**Figure 5:**
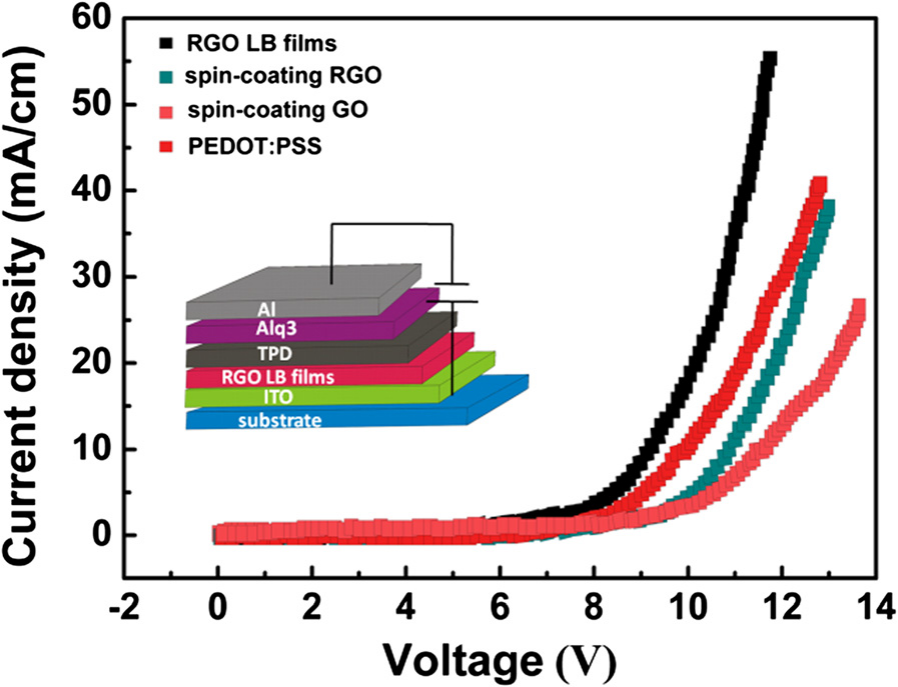
**Current-voltage characteristic curves of OLEDs with different films.** Current-voltage characteristic curves of OLEDs with different films as hole injection layer. Inset: illustration of device structure based on RGO LB films.

**Figure 6:**
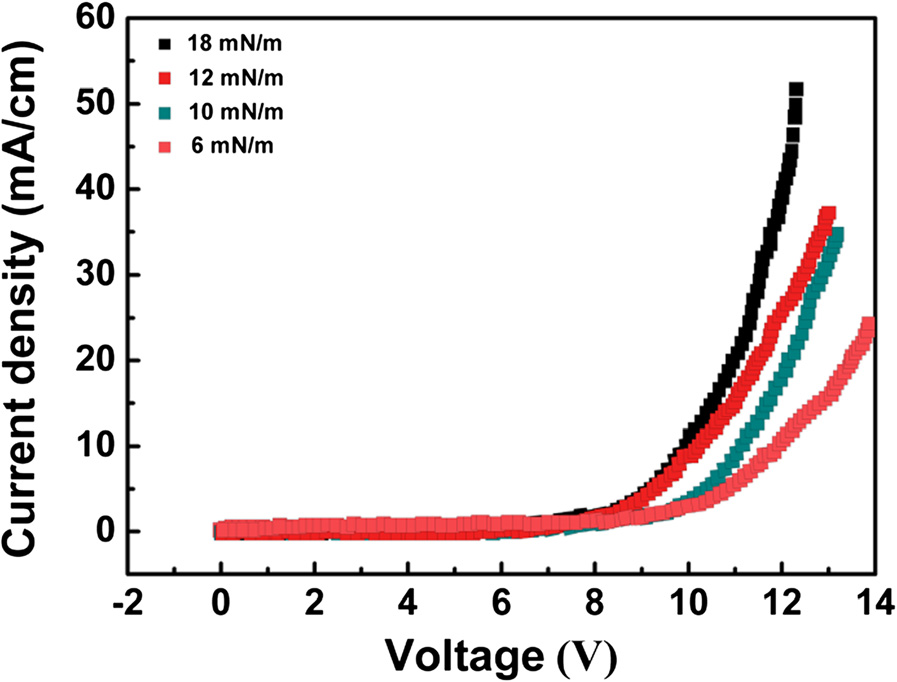
Influence of film deposition pressure on luminance-voltage performance of devices.

The thermal treatment results in the change of structure and conductivity performance of GO, leading to the formation of RGO with better conductivity. As shown in Figure 3, the I-V characteristics of GO and RGO LB films display a dramatic enhancement of conductivity after the thermal treatment, indicating the successful thermal reduction of GO to RGO. The thermal treatment makes the reduced GO resemble graphene, whose conductivity is comparable to that of doped conductive polymers, but with some residual oxygen and structural defects [12]. The linear I-V curves of GO and RGO LB films confirm the good ohmic contact between LB film and electrodes. The electrical resistance of GO LB films treated at 200°C is about 5.2 × 10^3^/(Ω m), which is higher than spin-coating PEDOT:PSS films (about 7.3 × 10^2^/(Ω m)) and spin-coating RGO films (about 2.6 × 10^3^/(Ω m)). It has been found that the electrical performance of RGO LB films shows strong dependence on surface pressure of GO film preparation, and the higher surface pressure causes better conductivity of RGO. We conclude that higher surface pressure addresses more compact and continuous arrangement of RGO sheets, resulting in a uniform and compact conducting channel for effective carrier transfer.The electroluminescence (EL) performance of a device with GO, RGO, and commercial PEDOT:PSS as injecting layer are shown in Figure 4. The result indicates that the EL peaks of all devices are located at 578 nm. This result means that the change of injection layer shows no influence on EL peak of an OLED device.Figure 5 shows the I-V curves of devices with different materials as hole injection layer. Compared with spin-coating RGO, GO, and PEDOT:PSS films, the enhanced current density of a device is observed after the insertion of RGO LB films as hole injection layer. The ordered and well-defined structure of hole injection layer plays a important role for effective hole transportation due to the hopping transfer of hole carrier in hole injection layers. Hence, a conducting and well-ordered RGO structure is suitable for this hole injection layers. RGO LB films ensure the enhancement of current and effective hole carrier injection because of highly ordered structure. Moreover, the insertion of ordered and well-defined nanostructure as hole injecting layer further enhances the exciton formation from hole-electron combination. The RGO LB film-based device exhibits larger current at the same applied voltage, indicating a lower driving voltage of device. Compared with RGO LB films, the device with spin-coating RGO layer as hole injection layer shows inferior current performance due to the non-ordered structure of this injection layer. But, compared with GO-based device, better current performance of device based on spin-coating RGO is achieved due to the higher conductivity of RGO.Figure 6 shows the influence of film deposition on I-V performance of devices. It has been found that higher film deposition pressure results in better I-V performance as well as larger current at same voltage. We deduce that RGO LB films obtained from higher surface pressure addresses more compact and continuous arrangement of RGO sheets, resulting in uniform and compact RGO sheets for effective carrier transfer. However, a loose arrangement of RGO sheets makes it difficult for effective hole transferring, and it would also make a negative effect on efficient hole-electron combination and luminance efficiency in OLED.

**Figure 7:**
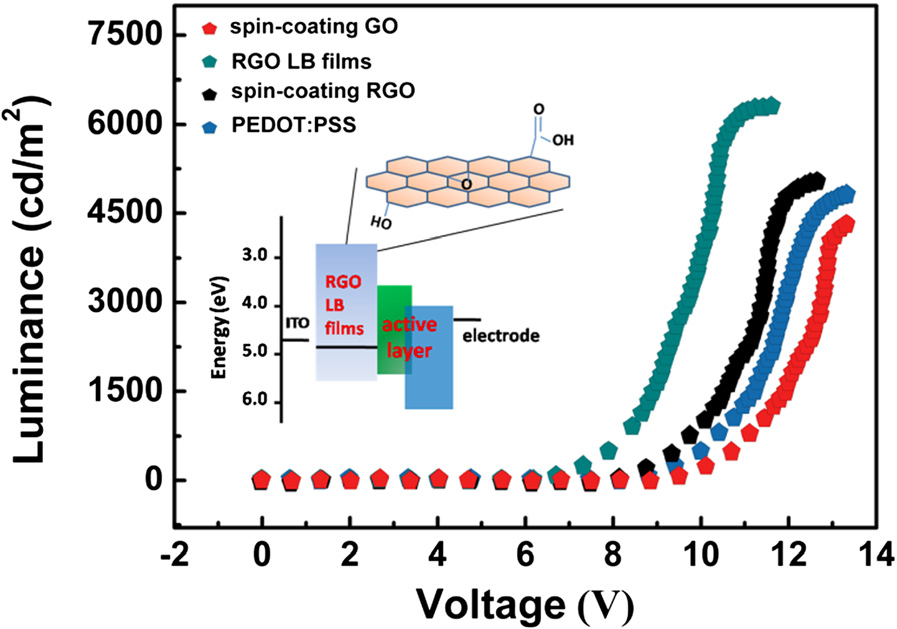
**Luminance-voltage characteristic curves of OLEDs with different hole injection layer.** Inset: energy level of different function layers in OLED.

Figure 7 shows the luminance-voltage performance of devices with different films as hole injection layers. The best luminance is achieved by device with the RGO LB films as hole injection layer. This device exhibits a maximum luminance of about 6,232 cd/m^2^ at a 12-V driving voltage, which is higher than the spin-coating RGO and commercial PEDOT:PSS devices. This result indicates that an ultrathin and well-ordered arrangement of nanosheets in hole injection layer obviously enhances hole injection efficiency in OLED, and this well-ordered hole injection layer is also important for OLED to achieve higher luminance performance. We also calculated the band energy of different functional layers in this device. The band energies of the ITO, TPD/Alq3, and Al layers are well known, and a detailed investigation shows that average work function (4.7 eV) of RGO thin films in our work is comparable to ITO for better hole injection.

Table 1 shows a detailed performance comparison of devices with different films as hole injection layer. Compared with other devices, the RGO LB film device achieves excellent performance in driving voltage, maximum luminance, and luminance efficiency. We also investigate the influence of heating temperature during reduction of GO on luminance performance of devices, which is shown in Table 2. It can be seen that, with the increase of heating temperature, the efficient reduction of GO is achieved and the RGO-based device exhibits improved luminance performance. In a word, we demonstrate the insertion of well-ordered and highly conductive RGO LB films as hole injection layer for OLED, and these RGO LB films could be an excellent alternative to commercial PEDOT:PSS as the effective hole injection and electron blocking layer for improving luminance efficiency.

**Table 1:** Performance of devices with different films as hole injecting layer

**Device**	**Driving voltage (V) for 100 mA/cm**^**2**^	**Max luminance (cd/m**^**2**^**, 12 V)**	**Luminance efficiency (cd/A, 12 V)**
PEDOT:PSS spin-coating film	11.7	4,435	2.9
GO spin-coating film	12.1	2,253	1.9
RGO spin-coating film	11.4	4,107	3.0
RGO LB film	9.6	6,232	3.8

**Table 2:** Influence of heating temperature during thermal reduction on device luminance performance

**Heating temperature (°C)**	**Driving voltage (V) for 100 mA/cm**^**2**^	**Max luminance (cd/m**^**2**^**, 12 V)**	**Luminance efficiency (cd/A, 12 V)**
50	11.3	4,015	2.5
150	10.1	5,527	3.3
200	9.7	6,144	3.7

## Conclusions

The LB assembly and a following reduction process produce the high-conductivity and well-ordered-structure RGO LB films. The results of I-V tests indicate that the thermal treatment changes the electrical performance of GO films dramatically. The RGO LB films are successfully incorporated between ITO and active layer as a hole injection layer. The incorporation of well-ordered and thickness-controlled RGO leads to an increase in recombination of electrons and holes as well as the block to the electrons. Our results indicate that RGO LB films are an excellent alternative to commercial PEDOT:PSS as the effective hole transport and electron blocking layer in OLED for improving luminance efficiency.

## Competing interests

The authors declare that they have no competing interests.

## Authors’ contributions

YY conceived of the study, carried out the fabrication of OLED, and drafted the manuscript. WY and XY helped to deposit LB films and analyze the data. YJ and JX helped to develop the idea and guided the study. SL helped to draft the manuscript. All authors read and approved the final manuscript.

## Authors’ information

WY and XY are students of a Master's degree at the School of Optoelectronic Information, University of Electronic Science and Technology of China. YY and SL are associate professors at the School of Optoelectronic Information, University of Electronic Science and Technology of China. JX and YJ are professors at the School of Optoelectronic Information, University of Electronic Science and Technology of China.

## References

[B1] Novoselov KS Faĺko VI Colombo L Gellert PR Schwab MG Kim K A roadmap for graphene Nature 2012 490 192 200 10.1038/nature11458 23060189

[B2] Geim AK Novoselov KS The rise of graphene Nat Mater 2007 6 183 191 10.1038/nmat1849 17330084

[B3] Bonaccorso F Sun Z Hasan T Ferrari AC Graphene photonics and optoelectronics Nature Photonics 2010 4 611 622 10.1038/nphoton.2010.186

[B4] Dan YP Lu Y Kybert NJ Luo ZT Charlie Johnson AT Intrinsic response of graphene vapor sensors Nano Lett 2009 9 1472 1475 10.1021/nl8033637 19267449

[B5] Borini S White R Wei D Astley M Haque S Spigone E Harris N Kivioja J Ryhänen T Ultrafast graphene oxide humidity sensors ACS Nano 2013 7 11166 11173 10.1021/nn404889b 24206232

[B6] Yang YJ Li SB Zhang LN Xu JH Yang WY Jiang YD Vapor phase polymerization deposition of conducting polymer/graphene nanocomposites as high performance electrode materials ACS Appl Mater Interfaces 2013 5 4350 4355 23621384 10.1021/am4003815

[B7] Wang B Su DW Park J Ahn H Wang GX Graphene-supported SnO_2_ nanoparticles prepared by a solvothermal approach for an enhanced electrochemical performance in lithium-ion batteries Nanoscale Res Lett 2012 7 215 221 10.1186/1556-276X-7-215 22500947 PMC3442962

[B8] Liu ZF Liu Q Huang Y Ma YF Yin SG Zhang XY Sun W Chen YS Organic photovoltaic devices based on a novel acceptor material: graphene Adv Mater 2008 20 3924 3930 10.1002/adma.200800366

[B9] Yang NL Zhai J Wang D Chen YS Jiang L Two-dimensional graphene bridges enhanced photoinduced charge transport in dye-sensitized solar cells ACS Nano 2010 4 887 894 10.1021/nn901660v 20088539

[B10] Pang SP Hernandez Y Feng XL Müllen K Graphene as transparent electrode material for organic electronics Adv Mater 2011 23 2779 2795 10.1002/adma.201100304 21520463

[B11] Fan XB Peng WC Li Y Li XY Wang SL Zhang GL Zhang FB Deoxygenation of exfoliated graphite oxide under alkaline conditions: a green route to graphene preparation Adv Mater 2008 20 4490 4493 10.1002/adma.200801306

[B12] Zhu YW Murali S Cai WW Li XS Suk JW Potts JR Ruof RS Graphene and graphene oxide: synthesis, properties, and applications Adv Mater 2010 22 3906 3924 10.1002/adma.201001068 20706983

[B13] Dreyer DR Park S Bielawski CW Ruoff RS The chemistry of graphene oxide Chem Soc Rev 2010 39 228 240 10.1039/b917103g 20023850

[B14] Chen D Feng HB Li JH Graphene oxide: preparation, functionalization, and electrochemical applications Chem Rev 2012 112 6027 6053 10.1021/cr300115g 22889102

[B15] Loh KP Bao QL Eda G Chhowalla M Graphene oxide as a chemically tunable platform for optical applications Nat Chem 2010 2 1015 1024 10.1038/nchem.907 21107364

[B16] Compton OC Nguyen ST Graphene oxide, highly reduced graphene oxide, and graphene: versatile building blocks for carbon-based materials Small 2010 6 711 723 10.1002/smll.200901934 20225186

[B17] Dong XC Huang W Chen P *In situ* synthesis of reduced graphene oxide and gold nanocomposites for nanoelectronics and biosensing Nanoscale Res Lett 2011 6 60 66 10.1007/s11671-010-9806-8 PMC3212207 27502682

[B18] Yin ZY Sun SY Salim T Wu SX Huang X He QY Lam YM Zhang H Organic photovoltaic devices using highly flexible reduced graphene oxide films as transparent electrodes ACS Nano 2010 4 5263 5268 10.1021/nn1015874 20738121

[B19] Gao Y Yip HL Chen KS O'Malley KM Acton O Sun Y Ting G Chen HZ Jen AK Surface doping of conjugated polymers by graphene oxide and its application for organic electronic devices Adv Mater 2011 23 1903 1908 10.1002/adma.201100065 21404333

[B20] Goki E Giovanni F Manish C Large-area ultrathin films of reduced graphene oxide as a transparent and flexible electronic material Nat Nanotechnol 2008 3 270 274 10.1038/nnano.2008.83 18654522

[B21] Tan LL Ong WJ Chai SP Mohamed AR Reduced graphene oxide-TiO_2_ nanocomposite as a promising visible-light-active photocatalyst for the conversion of carbon dioxide Nanoscale Res Lett 2013 8 465 673 10.1186/1556-276X-8-465 24195721 PMC3827867

[B22] Cote LJ Kim F Huang JX Langmuir-Blodgett assembly of graphite oxide single layers J Am Chem Soc 2009 131 1043 1049 10.1021/ja806262m 18939796

[B23] Kim JY Cote LJ Kim F Yuan W Shull KR Huang JX Graphene oxide sheets at interfaces J Am Chem Soc 2010 132 8180 8186 10.1021/ja102777p 20527938

[B24] Wen JF Jiang YD Yang YJ Li SB Conducting polymer and reduced graphene oxide Langmuir-Blodgett films: a hybrid nanostructure for high performance electrode applications J Mater Sci: Mater Electron 2014 25 1063 1071 10.1007/s10854-013-1687-z

[B25] Hummers WS Offeman RE Preparation of Graphitic Oxide J Am Chem Soc 1958 80 1339 1339 10.1021/ja01539a017

